# Characteristics of the Patients Aged Less than 40 Years Operated for a Renal Mass

**DOI:** 10.15586/jkcvhl.v10i4.287

**Published:** 2023-12-21

**Authors:** Abdullah Ilktac, Bayram Dogan, Cevper Ersoz, Ganime Coban, Senad Kalkan

**Affiliations:** 1 Department of Urology, Faculty of Medicine, Bezmialem Vakif University, Fatih, Istanbul, Türkiye;; 2 Department of Pathology, Faculty of Medicine, Bezmialem Vakif University, Fatih, Istanbul, Türkiye

**Keywords:** benign renal neoplasm, hereditary renal cell carcinoma, kidney tumor, renal cell carcinoma, young adults

## Abstract

The incidence of renal cell cancer (RCC) is low in individuals aged less than 40 years; however several studies have shown this increasing trend over the years. Hereditary syndromes are associated with RCC and are more frequently observed in early-onset cases. In this study, we investigated the characteristics of the patients, aged less than 40 years, who were operated for a renal mass with the suspicion of RCC. We analyzed patients aged <40 years who underwent partial or radical nephrectomy between January 2012 and December 2022. A total of 618 patients underwent partial or radical nephrectomy and 60 (9.7%) patients were aged <40 years. A total of 62 renal masses were resected. The median age of the patients was 34 (31.75–38) years. RCC was detected in 50 (80.6%) lesions, while 12 (19.4%) lesions were benign. The most commonly observed benign tumors were oncocytoma and multicystic nephroma. Low-stage RCC (stage 1) was detected in 78% of patients. Recurrence was observed in two patients and both had *von Hippel–Landau* gene mutation. During follow-up, two patients were found to have lung metastasis, while another patient had bone metastasis. Three patients died during the follow-up period. Disease free survival rate was 89.58% and cancer specific survival rate was 93.88%. The incidences of kidney cancer in young adults are increasing; therefore, early discovery and the diagnosis are important. Further research is required to gain a better understanding.

## Introduction

Kidney cancer is the 14th most common cancer in the world and it is more frequently observed in men than in women (1). However, detection rate has increased recently due to the widespread use of abdominal imaging modalities. Renal cell carcinoma (RCC) constitutes 90% of kidney cancer patients. The incidence of RCC increases with age (2). The median age at the time of diagnosis varies across countries, with the United States reporting a median age of 64 years (1). Siegel et al. reported that the majority of observed patients were aged more than 60 years (3). In this study, our objective was to assess the characteristics of RCC among young adults. Although various age thresholds have been utilized in the literature to differentiate between young and older patients, the predominant criterion defining young patients in most studies is 40 years of age (4–6). It is important to recognize that factors influencing the behavior and biology of RCC, as well as survival, differ in young patients. Hereditary syndromes, such as von Hippel–Landau (VHL), Birt–Hogg–Dube, and tuberous sclerosis, are associated with RCC and are more frequently observed in early-onset cases (7). Although the incidence of RCC is generally low in individuals aged less than 40 years, comprising 3–7% of all RCC cases, several studies have shown an increasing trend over the years (4, 8, 9).

The increase in the incidence of RCC is primarily attributed to the improved detection of renal masses through abdominal imaging techniques, such as ultrasonography, computed tomography (CT), and magnetic resonance imaging (MRI) (10, 11). However, it should be noted that a significant proportion of these masses initially diagnosed as RCC based on imaging findings were ultimately determined as benign lesions upon pathological examination. The limitations of CT and MRI in accurately differentiating between malignant and benign tumors, such as oncocytoma and fat-poor angiomyolipoma (AML), have been highlighted in several studies (12–14). Bauman et al. reported that out of 916 patients who underwent partial nephrectomy with the initial diagnosis of RCC, 14.1% had benign lesions upon pathological examination following the surgery (15).

According to the Bosniak classification, cystic lesions of the kidney are classified into five categories based on CT imaging (16). Approximately half of the Bosniak 3 cysts and 83% of the Bosniak 4 cysts are malignant (17). Surgery is considered over treatment in 60% Bosniak 3 and Bosniak 4 cyst patients.

In this study, we investigated the characteristics of the patients, aged <40 years, who were operated for a renal mass with the suspicion of RCC, and determined the ratio of patients with a benign pathological diagnosis after surgery.

## Material and Methods

After obtaining approval from the Institutional Ethics Committee (approval No.: 2023/167), we conducted a retrospective evaluation of clinic database. We analyzed patients who underwent partial or radical nephrectomy between January 2012 and December 2022 because of a renal mass suspected as being RCC. Prior to surgery, all patients underwent a comprehensive physical examination and laboratory tests, including renal function tests, as well as CT or MRI. Surgery was recommended for patients with a suspicious solid renal mass or those with a Bosniak 3 or Bosniak 4 cystic lesion at our institute. The type (partial/radical) and technique (open/laparoscopic) of the surgery were determined according to the size and location of the lesion and preference of both surgeon and patient. Patients aged ≥40 years or <18 years, with no abdominal imaging or pathological report, patients with the preoperative diagnosis of a benign lesion, and those diagnosed with urothelial carcinoma were excluded from the study.

Age at the time of surgery, gender, comorbidities, type of surgery (partial vs. radical), surgical technique (open vs. laparoscopic), laterality, clinical manifestations at presentation, size of the lesion (measured as the longest diameter during pathological examination), pathological diagnosis, multifocality, Fuhrman grade, pathological stage, surgical margin status, and duration of follow-up were recorded and analyzed in this study. Readmission to the clinic or emergency service within 30 days following the surgery was documented. The pathological evaluation was conducted based on the tumor node metastasis (TNM) classification (18). The presence of metastasis, recurrence, reoperation, and mortality proportions during the follow-up period were determined. The follow-up period was defined as the time from operative surgery up to patient’s death, or the last recorded follow-up. Informed consent was obtained from all the patients, and this study was conducted according to the principles of the Declaration of Helsinki.

## Statistical Analysis

Data storage and statistical analyses were performed using the Statistical Package for the Social Sciences (SPSS) 24.0 statistical program (SPSS Inc., Chicago, IL, USA). Descriptive statistics, including numbers, proportions, medians, and interquartile range (IQR) values were calculated. Data were tested to investigate whether they were normally dispersed using visual (probability plots and histograms) and the Kolmogorov–Smirnov test. Chi-square and Fisher’s exact tests were used to evaluate the association between dichotomous categorical variables. The Mann–Whitney U test was used to compare differences between two independent groups. Statistical significance was defined as P < 0.05.

## Results

In our clinical database, 618 patients underwent surgery for a suspicious renal mass between January 2012 and December 2022. Among these patients, 60 (9.7%) were aged less than 40 years. Number of patients in the age groups of 40–49, 50–59, 60–69, and ≥70 years were 126 (20.4%), 175 (28.3%), 164 (26.5%), and 93 (15.1%), respectively. All the detected masses were unilateral. A total of 62 masses were resected, as two patients had multiple lesions in the same kidney. In one patient, both lesions were diagnosed as RCC, while in the other, one lesion was identified as an oncocytoma and the other as a papillary adenoma. None of the patients underwent preoperative renal mass biopsy. The demographic data of the patients aged <40 years are presented in Table 1. The median age of the patients was 34 (31.75–38) years. The median lesion size was 44 (29.25–60) mm. Partial nephrectomy was performed in 44 (73.3%) patients and radical nephrectomy was performed in 16 (26.7%) patients. Most of the patients were asymptomatic.

**Table 1:** Demographic data of patients.

	N	%
Number of patients	60	
Number of lesions	62	
Gender		
Male	35	58.3
Female	25	41.7
Symptoms		
Incidental	42	70
Hematuria	6	10
Flank pain	12	20
Type of surgery		
Partial nephrectomy	44	73.3
Radical nephrectomy	16	26.7
Technique		
Open	44	73.3
Partial	35	
Radical	9	
Laparoscopic	16	26.7
Partial	9	
Radical	7	
	**Median**	**IQR**
Age (years)	34	31.75–38
Size of the mass (mm)	44	29.25–60
Operation time (minutes)	123	112.5–180

IQR: Interquartile range.

Of the 62 lesions, RCC was detected in 50 (80.6%) lesions in 49 patients, while 12 (19.4%) lesions were benign. The most commonly observed benign tumors were oncocytoma and multicystic nephroma (Table 2). Among the patients, 45 (75%) had solid masses detected in the kidney, while 15 (25%) had cystic masses. Of these 15 patients, nine had Bosniak 3 cysts and six had Bosniak 4 cysts. All patients with Bosniak 4 cysts were ultimately diagnosed with RCC after pathological examination. Among patients with Bosniak 3 cysts, six patients (66.7%) were diagnosed with RCC, while the remaining three patients had multi-cystic nephroma, simple renal cyst, and papillary adenoma. The median tumor size for RCC and benign tumor was 44 (29.25–60) mm and 45 (28.5–60) mm, respectively (P = 0.342). The majority of patients diagnosed with RCC had low-stage cancer, with 78% of them being classified as having stage T1 cancer (Table 3). However, one patient had metastatic disease at the time of surgery, with multiple metastases observed in the lungs, mediastinal area, hilar region, and supraclavicular lymph nodes. In this patient, an 80-mm mass was present in the kidney, and a cytoreductive surgery was performed. The pathological examination revealed an unclassified RCC. Gender had no effect on the detection rate of RCC and benign tumors (Table 4). Furthermore, no disparity was found in the detection proportion of RCC and benign tumors while comparing lesions ≤40 mm to lesions >40 mm, as shown in Table 5.

**Table 2:** Pathology results of 62 masses from 60 patients.

Renal Cell Cancer, n (%)	50 (80.6)
Clear cell	40
Papillary	3
Chromophobe	4
Other	3
Oncocytoma, n (%)	3 (4.9)
Multicystic nephroma, n (%)	3 (4.9)
AML, n (%)	2 (3.2)
Simple cyst, n (%)	2 (3.2)
Benign tissue, n (%)	1 (1.6)
Papillary adenoma, n (%)	1 (1.6)

AML: angiomyolipoma.

**Table 3:** Characteristics of 49 patients with RCC.

Number of patients	49
Number of lesions	50
Median age (IQR), years	34 (31.75–38)
Gender, n (%)	
Male	31 (63.3)
Female	18 (36.7)
Pathological stage, n (%)	
pT1a	25 (50)
pT1b	14 (28)
pT2a	2 (4)
pT2b	2 (4)
pT3a	4 (8)
pT3b	1 (2)
pT3c	1(2)
pT4	1 (2)
Fuhrman grade, n (%)	
1	2 (4)
2	24 (48)
3	15 (30)
4	3 (6)
Not available	6 (12)
Positive margin, n (%)	3 (6)
N+, n (%)	1 (2)
M+, n (%)	1 (2)
Early readmission rate (%)	6.67
Median follow-up (IQR), months	57 (35.75–76.5)

N+: presence of metastatic lymph node; M+: presence of metastasis.

**Table 4:** Comparison of RCC and benign tumor detection rate according to gender.

	Female (n: 25)	Male (n: 35)	P*
RCC (%)	18 (72)	31 (88.6)	0.174
Benign (%)	7 (28)	4 (11.4)	

RCC: renal cell carcinoma

* Fisher’s exact test.

**Table 5:** Comparison of RCC and benign tumor detection rate according to lesion size.

	Lesion size ≤ 40 mm	Lesion size > 40 mm	P*
RCC, n (%)	25 (83.3)	25 (78.1)	0.423
Benign, n (%)	5 (16.7)	7 (21.9)	

RCC: renal cell carcinoma.

* Chi-square test.

The decision regarding genetic testing was taken by the patient’s physician. However, we are unable to provide the exact number of patients who underwent genetic testing. Among the entire patient cohort, three patients exhibited a *VHL* gene mutation, one patient presented with Xp11.2 translocation RCC involving *TFE3* gene rearrangement, and another was diagnosed with succinate dehydrogenase (SDH)-deficient RCC because of a germline mutation in the *SDHB* gene

The median follow-up period was 57 (35.75–76.5) months. Patients were monitored according to the recommended follow-up schedule outlined in the European Urology Association Renal Cancer Guidelines (19). Recurrence was observed in two patients who underwent partial nephrectomy. Both patients had *VHL* gene mutation, and a re-partial nephrectomy was performed as an approach treatment. In another patient, suspicious lesions, measuring <3 cm in diameter, were identified on imaging; the patient is currently under active surveillance. In one patient who underwent radical nephrectomy, a 20-mm paracaval lymph node was resected 6 months after the surgery. Pathological examination confirmed the presence of RCC metastasis in the lymph node. This patient declined further oncological treatment, and during the 51-month follow-up period, no metastasis or local recurrence was detected. During the follow-up period, two patients, who underwent radical nephrectomy, were found to have lung metastasis, while another patient had bone metastasis. No metastases were detected in patients who had partial nephrectomy. All patients of partial nephrectomy had negative surgical margins, indicating successful removal of tumor with clear margins. On the other hand, three patients of radical nephrectomy had positive surgical margins, but no metastasis or local recurrence occurred in these patients. Three patients died during the follow-up period. One of these patients underwent cytoreductive nephrectomy. Disease-free survival rate was 89.58% and cancer-specific survival was 93.88%. The readmission rate of 1 month after surgery was 6.7% for the entire group. Angioembolization was performed in a patient who presented with hematuria at postoperative 3rd week. Another patient developed urinoma 2 weeks after the surgery, and percutaneous drainage was performed along with the placement of a double J catheter. Two patients who underwent radical nephrectomy experienced hematoma formation. Of these, one patient underwent exploration, while the other was managed conservatively. All patients readmitted within 30 days after discharge had RCC. No death was reported in the perioperative or early 30-day postoperative period.

The available data about 558 patients aged ≥40 years who underwent renal mass surgery in our clinic were limited, yet significant differences were discovered compared to younger patients. Notably, the number of patients who underwent radical nephrectomy was higher among individuals aged ≥40 years (40.1% vs. 26.7%; P = 0.02). RCC was the most prevalent kidney tumor in patients aged ≥40 years, with the clear cell subtype being the most common presence, similar to the pattern observed in younger patients. Conversely, multicystic nephroma was less frequently observed in patients aged ≥40 years, compared to their younger counterparts (4.9% vs. 1.8%; P = 0.03). Younger patients more frequently presented with pT1a diseases, compared to those aged ≥40 years (50% vs. 39.8%; P = 0.006), although older patients showed a higher prevalence of pT3a stage (23.9% vs. 8%; P = 0.001). No significant differences were observed among other stages. The proportion of patients aged ≥40 years with positive surgical margins and lymph node invasion was 6.3% and 2.2%, respectively, with no observed statistically significant differences. However, our dataset had no information regarding the follow-up period, metastasis, and genetic testing outcomes for patients aged ≥40 years.

## Discussion

Although kidney masses are more common in the elderly population, they can also be observed in young adults aged <40 years. The results obtained from the limited number of studies conducted in this age group were mostly similar to those in elderly patients. The incidence of metastatic disease is very low, and the majority of patients present with low-stage renal tumors. Additionally, a few patients may have benign tumors. Notably, in most cases, renal masses are detected incidentally during abdominal imaging performed for other reasons. Therefore, even in young individuals, it is crucial to consider the presence of a renal mass during abdominal imaging.

Yikilmaz et al. evaluated 129 patients who underwent nephrectomy due to suspected malignancy; of these, six (4.6%) patients were aged <40 years (20). In a series including 514,849 patients diagnosed with RCC, Douglas et al. reported that 4.7% of the patients were aged <40 years (21). Kang et al., in their study involving 5178 patients with RCC, discovered that 10.45% of the patients were aged <40 years (5). In the current study, the proportion of patients aged <40 years operated due to a suspicious mass was 9.7%. It was slightly higher, compared to the proportions reported in the literature but closer to that reported by Kang et al. (5).

Computed tomography and MRI were commonly used for the characterization of renal masses (22). In most cases, these radiological imaging methods successfully diagnosed RCC, but occasionally they faced challenges in differentiating oncocytoma and lipid-poor AML from RCC (12–14). In the general population, the detection rate of benign tumors in patients who had surgery for RCC was reported as being around 11–16% (23–25). Aslan et al. conducted a study evaluating localized renal masses in patients aged <40 years, and reported that 24% of the patients were diagnosed with benign lesions (26). According to Yikilmaz et al., a benign lesion was discovered during pathological examination in 16% of the patients aged <50 years (20). Our findings were consistent with the literature. We observed that 18.3% of the patients who underwent surgery for RCC had benign tumors discovered during pathological examination. Among these, oncocytoma, AML, and multicystic nephroma were the most frequently discovered benign tumors.

Several studies conducted on young adults reported similar results regarding the types of benign lesions (23, 24, 26). The diagnosis of RCC and the decision for surgery primarily depended on radiological imaging methods. Renal tumor biopsy was proposed to prevent overtreatment and unnecessary surgeries in individuals with small renal masses (27, 28). Studies reported a diagnostic rate and concordance with surgical pathology of over 90% (27, 29). Although not widely accepted, renal tumor biopsy was used predominantly in metastatic diseases and prior to ablation therapy; however, there is a growing trend in its utilization for small renal masses (30). The identification of post-surgical benign lesions in approximately 20% cases emphasizes a significant issue, suggesting that some patients undergo unnecessary surgical interventions. Renal tumor biopsy could potentially avert these unnecessary interventions.

Pathological stage is the most crucial factor influencing the prognosis of patients with RCC, with a worse prognosis associated with higher stages (19). In this study, 78% of the patients with RCC were diagnosed with stage 1 cancer. Gillet et al. conducted a comparative study between patients aged 18–40 years and 60–70 years, revealing that individuals aged <40 years were more likely to have a low-stage tumor (6). Douglas et al. reported that patients aged ≤40 years had a higher frequency of low-stage tumors and a lower rate of metastatic disease, compared to patients aged >40 years (21). Kang et al. stated that RCC patients aged <40 years had a lower tumor stage, a higher rate of favorable pathological features, and better cancer-specific survival (CSS) (5). Aziz et al. found that patients aged between 60 and 70 years had significantly higher disease-specific and overall mortality, compared to the patients aged ≤40 years (31). However, Gillett et al. (6) and Thompson et al. (24) did not observe a difference in CSS among patients in different age groups. Taccoen et al. reported that age was an independent risk factor for survival (32).

In this study, clear cell RCC was found as being the most commonly observed subtype, accounting for 40 (80%) of the 50 lesions in 49 patients with RCC. Papillary cell RCC and chromophobe cell RCC were detected in 6% and 8% patients, respectively. Kang et al. reported the proportions of clear cell, papillary cell, and chromophobe cell RCCs in young adults as 82.3%, 6.3%, and 7%, respectively (5). Douglas et al. stated that 55.2% of the patients aged ≤40 years had clear cell RCC. When evaluating non-clear cell RCC patients, they found that younger patients were more likely to have chromophobe RCC, while in patients aged >40 years, papillary RCC was observed more frequently than chromophobe RCC (21). Other studies in the literature also reported that chromophobe RCC was the most common non-clear cell subtype in young adults (6). In our study, we observed that chromophobe RCC was the most common non-clear cell RCC subtype which aligns with the findings of the literature.

Size was a significant factor in predicting the nature of renal masses. Several studies have reported that increasing tumor size is associated with malignancy, with a higher incidence of benign tumors observed in smaller masses (33). Bhindi et al. stated that patients with masses measuring 2 cm, 3 cm, and 4 cm had a likelihood of 84%, 85%, and 87%, respectively, of having malignant tumors (25). However, these studies were conducted in the general population, and there were no similar studies in the literature specifically focused on younger adults. In our study, we found no significant difference in mean tumor size between patients with benign tumors and those with RCC. Aslan et al. also reported similar mean tumor sizes in young adults with benign and malignant masses (26). Furthermore, studies have shown that the probability of observing a benign lesion after histopathological evaluation in patients undergoing surgery for RCC is higher in women. Aslan et al. reported 35% of young women having benign lesions after surgery, while this rate was only 13.6% in men, and this difference was statistically significant (26). Similarly, Yikilmaz et al. also reported a significantly higher rate of benign lesions in young women (20). In our study, we found no difference in detection rate of benign tumors in men and women.

Some patients with early-onset RCC may have an underlying hereditary predisposition manifesting as multiple or bilateral tumors (34). Shuch et al. recommended that genetic testing and counselling should be conducted in patients aged ≤46 years with RCC, even in the absence of clinical manifestations or family history (7). Some of the most common hereditary syndromes associated with RCC include VHL, Birt–Hogg–Dube, and tuberous sclerosis (34). In our series, we identified three patients with *VHL* gene mutation. All these patients experienced recurrence, with two of them undergoing partial nephrectomy and one patient being closely monitored through active surveillance because of the small size of lesions (<30 mm). Additionally, we had a patient with Xp11 translocation RCC and another with SDH-deficient RCC. Xp11 translocation RCC is a rare cancer in adults but is frequently observed in children and adolescents, constituting 20–75% of renal neoplasms in the paediatric population (35). This subtype is characterized by chromosome translocations involving the *TFE3* gene and it is an invasive tumor with a poor prognosis (36). In our study, the patient with Xp11 translocation RCC presented with a 110-mm tumor and a tumor thrombus extending to the inferior vena cava. The pathological stage was pT3bN0. During the 1-year follow-up period, no metastasis or recurrence was discovered. SDH-deficient RCC, on the other hand, accounts for 0.05–0.2% of all RCC cases and is primarily observed in young adults (37). Low-grade tumors with favorable histological features generally have a better prognosis, with a low risk of metastasis. Conversely, tumors with high-grade nuclei and sarcomatoid dedifferentiation tend to have a more aggressive course and a higher risk of metastasis (37). In our study, the patient presented with a favorable low-grade tumor. The tumor size was 18 mm, and the patient underwent a partial nephrectomy. Pathological staging revealed a pT1aN0M0 tumor, indicating a localized disease. Pathological evaluation confirmed a low-grade tumor with no dedifferentiation. Importantly, no recurrence or metastasis was observed during the 50-month follow-up period.

This study had several limitations that must be acknowledged. First, it was a retrospective study with a limited number of patients, which could limit the generalizability of results. The data included all patients aged <40 years who underwent surgery for a suspected RCC mass, including one patient with metastatic disease. However, patients with RCC who were not operated for various reasons, such as those referred for systemic therapy, were not included in the study. Additionally, genetic testing and evaluation were not conducted on all patients, which could have impacted the identification of underlying hereditary predispositions and the characterization of specific subtypes of RCC.

## Conclusion

Kidney cancer in young adults presents unique characteristics. Although it is less common, its incidence in young adults is increasing, highlighting the importance of early detection and the diagnosis. Our study contributes to the existing literature by providing insights into the clinicopathological features and outcomes of kidney cancer in this specific age group. Further research with larger cohorts and comprehensive genetic evaluations is endorsed to gain a better understanding of the underlying mechanisms and improve patient management in young adults with kidney cancer.
